# Biophysics in the time of COVID

**DOI:** 10.1007/s12551-022-00932-y

**Published:** 2022-02-09

**Authors:** Adam P. Hill

**Affiliations:** 1 Victor Chang Cardiac Research Institute, 405, Liverpool Street, Darlinghurst, Sydney, NSW 2010 Australia; 2 St. Vincent’s Clinical School, UNSW Sydney, Sydney, Australia

In March 2020 the WHO announced that COVID-19, the disease caused by the severe acute respiratory syndrome coronavirus 2 (SARS-CoV-2), had become a pandemic. At that time, around 118,000 cases had been reported. Some 2 years, 280 million cases and 5.4 million deaths later, COVID-19 has swept around the globe to dramatically change the way we live and work. The impact on scientific societies such as the ASB has been twofold. First, it has interrupted the academic careers of our members, through lockdown-enforced disruption to research and teaching as well as shifting their focus to meet the scientific challenges posed by the pandemic. Second, it has affected the day-to-day operations of the society, most significantly in relation to the annual scientific meeting, through limitations on travel and face to face contact.

On the first of these points, COVID-19 undoubtedly presented some significant hurdles to research. However, it has also seen scientists across disciplines pivot their efforts to help build our understanding of SARS-CoV-2 transmission and infection, develop low cost, accurate tests and identify new therapeutics. One thing that has become clear is the key role that biophysical research has to play in addressing these challenges. Soon after the SARS-CoV-2 genome was published at the beginning of 2020, the X-ray structure of the virus’s main protease was published (Jin et al. 2020), and this was soon followed by the cryo-EM structure of the spike protein (Wrapp et al. 2020) that has since become the target for the successful mRNA-based vaccines that have been rolled out around the world. As the pandemic has continued over the past 2 years, so too have the discoveries in the field of biophysics that will continue to help us address the ongoing challenges of COVID-19.

On the second issue—of society operations—our 44th annual meeting was scheduled to be held on the Gold Coast in 2020 but was cancelled as a result of Australia’s first wave of COVID-19. Instead, like many societies around the world, we made the decision to host a virtual meeting. While accepting that the online option perhaps comes at the cost of the community, networking and comradery of an in-person meeting, we looked to maximise the benefits from the virtual format to our members, particularly in terms of ECR engagement and international accessibility. By these measures, the meeting was extremely successful, with 180 registrants enjoying more than 60 oral presentations, 40% of them delivered by early career or student members. Attendees joined these sessions from countries across the globe including Australia, New Zealand, Iran, Saudi Arabia, Japan, USA, China, Singapore and South Korea—adding a truly global flavour. Over the 3 days of the meeting, we incorporated several themed sessions into the schedule. First amongst these was a new initiative for the ASB, a joint ECR symposium with the UC Davis Biophysics student chapter. Modelled on the Gordon Research Symposium concept, this was organised by an ECR committee, mentored by Dr Amanda Buyan from ASB and Dr Katelyn Jarvis from Davis, and was a fitting showcase for the outstanding ECR work done across our two societies. This was an excellent initiative, facilitated by the online format, that I hope we can incorporate into future hybrid scientific meetings. Also on the program were collaborative sessions with our regional partners. For 15 years now, the ASB has had an agreement with the Biophysical Society of Japan (BSJ) to incorporate joint symposia into our annual meetings. We were able to maintain this tradition in the virtual format and welcome four speakers from BSJ. This session attracted strong attendance from our Japanese colleagues that is also reflected in numerous contributions from BSJ members in this special edition. 2020 also saw us strengthen our ties with New Zealand, with election of Dr David Baddeley (University of Auckland) as our New Zealand Representative. To build on this relationship we added an ASB New Zealand section session to the program, comprising six talks from NZ researchers. I look forward to seeing this relationship flourish to grow our membership across the Tasman. Finally, in another first for the ASB scientific meeting, we held a session on careers for biophysics graduates. As part of this we were lucky enough to have experts from diverse fields including business strategy, patent law, scientific policy and academic biophysics speaking on their career experiences in an extremely popular session that was a valuable educational resource for our members in these uncertain times.

Overall then, the COVID-enforced virtual format was a positive experience for our scientific meeting with clear benefits of low cost, ease of access, increased ECR engagement, international contribution and reduction in our carbon footprint. While everyone is no doubt eager to return to physical meetings, I see it as likely that some online component will be a permanent fixture for our society operations, whether part of a hybrid format, as we undertook for the 45th meeting in 2021, or perhaps as a vehicle to facilitate state-based satellite meetings throughout the year. I look forward to reading more about the excellent science presented at the 44th meeting of the ASB in this special issue as well as the continued contribution of biophysics in Australia and around the globe in tackling the ongoing COVID-19 pandemic.
